# Near and Dear? If animal welfare concepts do not apply to species at a great phylogenetic distance from humans, what concepts might serve as alternatives?

**DOI:** 10.1017/awf.2024.36

**Published:** 2024-09-30

**Authors:** Saskia S Arndt, F Josef van der Staay, Vivian C Goerlich

**Affiliations:** 1 Division of Animals in Science and Society, Animal Behaviour Group, Faculty of Veterinary Medicine, Utrecht University, PO Box 80166, 3508 TD Utrecht, The Netherlands; 2 Department of Population Health Sciences, Division of Farm Animal Health, Behaviour and Welfare Group (Formerly: Emotion and Cognition Group), Faculty of Veterinary Medicine, University Utrecht, Utrecht, The Netherlands; 3 University Medical Center (UMC) Utrecht, Brain Centre, Utrecht, The Netherlands

**Keywords:** affective state, animal integrity, guidance for action, integrity of habitat and ecosystem, precautionary principle, sentience

## Abstract

A wide range of animal taxa, including vertebrates and invertebrates, are controlled or kept by humans. They may be used as pets, for recreation, sport and hobbies, as working animals, as producers of animal-derived (food) products or as biomedical models in research. There is a need for clear guidance on the treatment of animals, regardless of their phylogenetic distance from humans. Current animal welfare concepts, which emphasise animal sentience and the ability of animals to experience negative or positive mental states, are limited in scope to a small proportion of the animal kingdom, as the vast majority of species are (currently) thought to lack sentience. We discuss four options for addressing the question of which basic concept(s) could be used to derive guidelines for the treatment of animal species, sentient or non-sentient: (1) alternative concepts tailored to specific groups of species; (2) ‘welfare’ concepts not presupposing sentience; (3) the precautionary principle; or (4) the concept of animal integrity. Since questions regarding the appropriate treatment of animals, including species with a large phylogenetic distance from humans, have an ethical/moral dimension, we also address who counts morally and how much, and how animals should be treated given their moral status. We suggest that the concept of animal integrity, possibly complemented and extended by the concept of habitat/ecosystem integrity, is suitable for application to all species. However, a current concept of animal welfare should serve as the primary basis for guidance on how to treat species that are sentient and capable of experiencing emotions.

## Introduction

Animal welfare and welfare management have received increasing public and academic attention in recent years (Bayvel & Cross 2010; Ohl & van der Staay 2012). Ethicists have been tasked with providing theoretical concepts of human obligations to the animals they keep, while animal scientists have been tasked with developing objective and quantifiable assessment tools for an animal’s welfare status under given conditions. Finally, policy-makers, together with animal scientists, have the responsibility to formulate guidelines for the treatment of animals based on scientific insights (Ingenbleek *et al.* 2012). Unfortunately, there is still much work to be done to reach a wider consensus on the assessment of an animal’s welfare status and the evaluation of the impact of the management and treatment of animals on their welfare status. All of these topics are subject of ongoing research.

In particular, the Five Freedoms concept (Brambell *et al.* 1965) and the Five Domains model (Mellor & Reid 1994) have strongly influenced contemporary animal welfare studies and have focused attention on factors considered relevant for measuring and improving animal welfare (Blokhuis *et al.* 2010; Hampton *et al.* 2023), albeit with varying degrees of specificity. These and other concepts provide the conceptual framework and theoretical basis for the development of policies and strategies to improve animal welfare, once translated into practical measurement tools and guides for action (Webster 2016). The formulation of animal welfare policies can thus be seen as a realisation of the animal welfare concepts.

In this paper, an animal is defined as a member of the kingdom Animalia that is a multicellular organism of well-defined form, usually of limited growth, capable of voluntary movement, actively acquiring food and digesting it internally, and possessing sensory and nervous systems that allow the organism to respond rapidly to stimuli. Although they are motile and have animal-like feeding habits, we do not include protozoa and other unicellular eukaryotes when referring to animals.

Animals are kept on farms (mammals such as cows, pigs, sheep, goats, rabbits; birds such as chickens, ducks, geese; fish such as salmon; Miller *et al.* 2022), or are caught in the wild (game, fish, crustaceans, molluscs, etc) for various purposes, such as human consumption (e.g. meat, milk, eggs), or the provision of animal-derived products (e.g. leather, wool, down and feathers, etc). Over the last decade, there has been a steady increase in the number of farmed insects for the production of human food (e.g. crickets; Magara *et al.* 2021) or animal feed (e.g. mealworms, black soldier fly larvae; Thrastardottir *et al.* 2021). Animals may serve as companions and pets and are also kept for recreation and sport (e.g. horse riding), in private households, stables, zoos, game reserves, safari parks and national parks. In biomedical research, several animal species are used as model species (e.g. vertebrates; Bähr & Wolf 2012; van der Staay *et al.* 2017, invertebrates; Wilson-Sanders 2011).

There is broad consensus that mammals, birds and fish are sentient, i.e. that they have the ability to experience pain and discomfort. Animal welfare concepts provide guidance on the treatment of sentient species, serving as a biologically based moral compass or, according to Webb (2019a), as an *animal ethics agenda* (Voogt *et al.* 2023). Nevertheless, *welfare* is a rather abstract term that is subject to redefinition, depending on changing scientific insights, societal opinions, and ethical considerations (Carenzi & Verga 2009; Ohl & van der Staay 2012; Englund & Cronin 2023). Ethicists and philosophers have considered the moral status of sentient and non-sentient animal species and the resulting moral obligations of humans towards these species. However, these reflections have mainly concerned animal species that are sentient and capable of experiencing emotions.

In order to reach a general consensus on animal welfare – its definition, its assessment, i.e. methods for quantifying welfare, and the resulting obligations for humans – a number of concepts and frameworks with different theoretical underpinnings have been developed. The welfare of intensively farmed animals has been the focus of animal scientists since the report of the Brambell Committee in the mid-1960s (Brambell *et al.* 1965), which formulated the Five Freedoms concept. This concept was further developed to provide a framework for the analysis of animal welfare (Farm Animal Welfare Council [FAWC] 1979).

Other frameworks of animal welfare followed, such as the Five Domains, originally formulated in the mid-nineties, addressing the impact of research procedures on the welfare of laboratory animals (Mellor & Reid 1994). The more recent Quality of Life concept, inspired by human psychology (Green & Mellor 2011) and medicine (in particular in relation to mental health), focuses on how the individual perceives its own welfare state.

The development of the science of animal welfare has focused primarily on (sentient) vertebrate species, i.e. animal welfare is thought to depend on an animal being sentient (i.e. capable of feeling emotions) (Bracke *et al.* 2023). However, concerns have been raised for decades regarding our lack of knowledge regarding the capacity of animal species more phylogenetically distant from humans to suffer, and the implications for how we treat these species (Sherwin 2001). For these species, the debate about sentience and their ability to experience (positive) emotions (Mellor 2019) continues, as scientific evidence for sentience is still scarce or absent (van Loon & Bovenkerk 2021). As a result, action-guiding knowledge for the appropriate treatment of (presumably) non-sentient species is still largely lacking. However, with regard to their treatment, a *moral compass* can be adopted that articulates our obligations towards non-sentient animals, ideally based on relevant scientific evidence (Gjerris *et al.* 2016).

Not least in view of the increasing use of invertebrate species in science and agriculture (e.g. farming of insects for food production; Baiano 2020), the question of whether (current) welfare concepts can be applied to these taxonomic classes (Mikhalevich & Powell 2020), or whether, and if so which, other concepts need to guide their treatment should be urgently addressed and answered. For example, De Goede *et al.* (2013) state that there is little scientific information available on how insects should be reared, let alone in relation to their welfare. In current welfare concepts, the ability to experience negative or positive mental states plays a central role (see above) (Mellor 2019). However, sentience is likely restricted to a small proportion of all living animals, who nearly all belong to the vertebrates (Titley *et al.* 2017). Therefore, these concepts of welfare may not be applicable to most other animal species, and one might even conclude that for species that are not sentient, *“we do not have to care about their welfare, as they do not have any welfare”* (Ng 2016; p 3). However, we may have moral obligations to these species for other reasons, as discussed below.

Welfare concepts provide the basis for formulating guidelines for how we should treat and interact with sentient species, i.e. these concepts have an action-guiding role. For non-sentient species, guidance is largely lacking because current welfare concepts are not applicable to them and are therefore irrelevant. We may therefore have to consider whether we need a more comprehensive, holistic view of the behavioural and mechanistic processes that may serve as guide for treatment of animals across a variety of taxa. In this paper, we draw attention to these upcoming challenges regarding guidelines for actions that comprise all animal beings, or, if necessary, concepts per taxonomic class. We explore different concepts that could serve as the basis for potential alternative guidelines for the treatment of (non-sentient) species with a large phylogenetic distance from humans, focusing on the precautionary principle and the concepts of integrity. Neither of these two concepts is new, but with the rapid growth of insect farming for food production, for example, the issue has become relevant again.

## The problem: The scope of application of current animal welfare concepts is limited to sentient species

In the following, concepts and definitions relevant to answering the question of the scope of current animal welfare concepts are reviewed and alternative approaches are discussed. At the heart of these concepts are often animal sentience and animal integrity. It should be noted that these two topics seem to be discussed more often in alternative, specialised journals, such as *Animal Sentience*, *Consciousness and Cognition* or in specialised animal welfare journals than in mainstream neuroscience or veterinary journals (an overview of relevant journals can be found, for example, at: https://www.animal-ethics.org/journals-specializing-in-animal-issues/; accessed January 17, 2024).

In addition to safeguarding unimpaired biological functioning, the inclusion of sentience and conscious mental states is increasingly recognised as a crucial necessity for good well-being (Boissy *et al.* 2007; Lerner 2008; Schmidt 2011; Veasey 2017; Mellor 2019). Current concepts of animal welfare apply only to sentient species, who are assumed to have the capacity to experience negative or positive mental states, at least those with a negative valence, such as pain. *“Definitions of animal welfare typically appeal to sentience, consciousness, experience, subjective feeling, or related ideas”* (Birch 2022; p 2). *“Modern views on animal welfare emphasize the role of animal sentience, i.e. the capacity to experience subjective states such as pleasure or suffering, as a central component of welfare”* (Browning & Veit 2022b; p 1). This view of welfare can be described as *zoocentric* (Schmidt 2011). Traditionally, the approach to animal welfare focused on *“unpleasant mental states in animals, such as pain, suffering, stress, distress, and discomfort”* (Tannenbaum 2002; p 24). Recently, the importance of including the presence of positive states, rather than just the absence of negative states, in defining and assessing welfare has been emphasised (Proctor *et al.* 2013; Mellor 2015). Thus, a crucial component of most recent concepts of animal welfare is the assumption that animals are able to experience negative or positive mental states, and that welfare is linked to experiencing positive emotions (e.g. Ohl & van der Staay 2012; Mellor & Beausoleil 2015; Webb *et al.* 2019b; Arndt *et al.* 2022). These concepts thus *“take a subjective, or hedonic, view of animal welfare, in which welfare consists in the subjective mental states experienced by an animal”* (Browning 2022; p 37).

The Dynamic Animal Welfare Concept (DAWCon) proposes the animal’s emotional state to have a central role:

*“An individual is likely to be in a positive welfare state if* [he or she] *is mentally and physically capable and has the ability and opportunity to* [respond appropriately] *to sporadic or* [sustained] *appetitive and adverse internal and external stimuli, events and conditions.* [Appropriate responses] *are elements of an animal’s normal behaviour. They* [enable] *the animal to cope with and adapt to the demands of the (prevailing) environmental circumstances and to reach a state that* [he or she] *perceives as positive, i.e. that evokes positive emotions.”* (slightly modified from Arndt *et al.* 2022; modifications between square brackets).

Different views exist as to how good welfare may arise; the concept of positive animal welfare (PAW) refers to four features, namely positive emotions, positive affective engagement (PAE), quality of life (QoL) and happiness (Lawrence *et al.* 2019). *Hedonic positive welfare* is based on motivation and preference being met, whereas a *positive welfare balance* results from negative experiences being outweighed by positive ones (Rault *et al.* 2020). Similar to the *cumulative experience* concept (Pickard and members of the Animal Procedures Committee 2013), the dynamic animal welfare concept proposes that welfare across a lifetime may depend on the net impact of appetitive and adverse internal and external factors, which should not exceed the limit of adaptability of an individual (Arndt *et al.* 2022). We do not agree with Novack *et al.* that “*welfare describes the state of an animal at one point in time*” (Novack *et al.* 2023; p 3). Instead, measurements at multiple time-points are required to infer the welfare state of an animal (Yeates 2016; Arndt *et al.* 2022).

The capacity of animal species being sentient and conscient, and experiencing emotions and affect has long been the subject of heated debate (Darwin 1872). Although most behavioural scientists will agree with the assertion that vertebrate species are sentient, there is still ongoing discussion about sentience in even the phylum Chordata, e.g. of fish (Segner 2012; Sneddon 2015; Mason & Lavery 2022) or reptiles and amphibians, although evidence in favour of their sentience is accumulating (Lambert *et al.* 2019, 2022a,b). Similarly, the question of whether animals, in particular species with a large phylogenetic distance from humans, can feel pain, has been discussed fiercely for a long time already. While some suggest that invertebrates can feel pain (Bateson 1991), others deny pain sensitivity, even in certain classes of vertebrates (e.g. fish; Key 2016).

Meanwhile, the discussion, which is partly focused on semantics, may hinder rather than advance the development of an appropriate concept providing guide for action (see the concept of ‘duty of care’; Mellor & Stafford 2001; Council on Animal Affairs 2012; Ohl & Putman 2014; Weary & Robbins 2019; Learmonth 2020) for species not covered by current welfare concepts. Note, that whereas invertebrates constitute more than 95% of the animal kingdom (Titley *et al.* 2017), the majority of welfare-related studies is almost exclusively directed at vertebrates (Carere *et al.* 2011). This neglect of species with greater phylogenetic distance from humans may be due to a cognitive-affective bias towards more familiar species and disgust reactions towards invertebrates (Mikhalevich & Powell 2020). Due to this bias and the focus on the ability to experience negative or positive mental states, the discussion of how to treat whole clades of species has received little attention.

**Figure 1.**
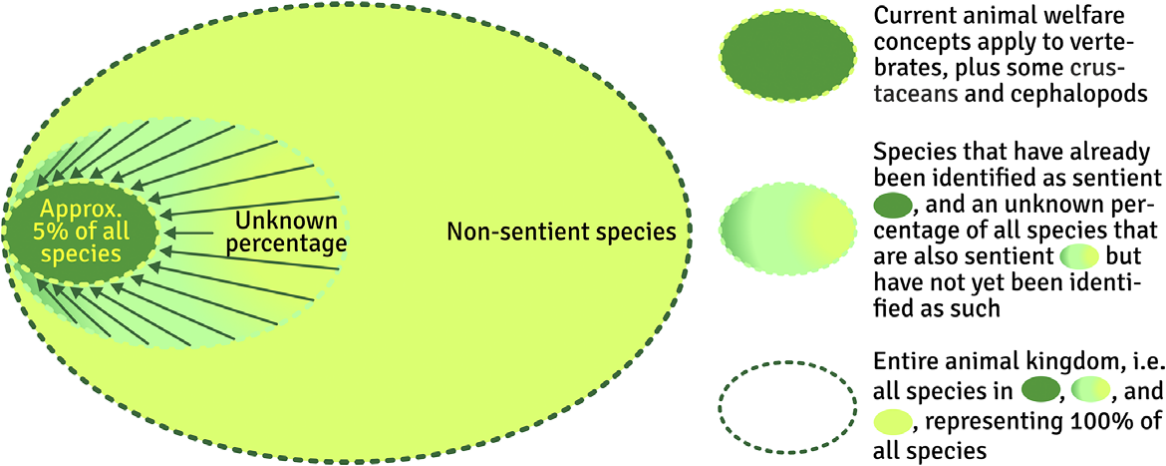
The problem – scope of applications of current welfare concepts.

It is very likely that for species with a large phylogenetic distance from humans, the condition of emotional capacity is not met or cannot be captured by current methods, due to increasing dissimilarities in species biology (Mendl *et al.* 2022). Consequently, concepts of animal welfare in which one of the criteria for good welfare is that an animal experiences (positive) emotions may be inapplicable or of limited relevance to most invertebrates. Alternative concepts are therefore needed for these taxa (see Figure 1).

Thinking about how to treat animals, whether sentient or non-sentient, also means that we have taken moral positions (implicitly or explicitly) about the ethical status of those animals and our moral obligations towards them. Humans are moral agents who ascribe moral status to animals, which means that humans should respect the interests and integrity of animals and be aware of how their actions may affect animals. Utilitarianism, an ethical theory, can provide a basis for motivating our moral obligations towards animals and for providing a moral compass for their treatment. Theories such as utilitarianism consider the moral rightness or wrongness of the outcome of actions, with the aim of “*producing the greatest happiness/good or least pain for the greatest number of individuals (which may include animals, based upon the moral status of animals)*” (Brown 2014; p 12), although some criticise utilitarianism as being too demanding when considering our obligations to animals (e.g. Hills 2009). Killoren and Streiffer note that “*utilitarianism (…) to involve two major commitments. First, utilitarians are welfarists about value: utilitarians believe that only states that constitute welfare are intrinsically (noninstrumentally) good or bad. Second, utilitarians are subjectivists about welfare: utilitarians believe that mental states either constitute welfare or determine which states constitute welfare*” (Killoren & Streiffer 2020; p 1050). This is reflected in current definitions of welfare by the importance of the ability to experience emotions and to reach a state that animals experience as positive (e.g. Arndt *et al.* 2022). In the case of sentient species, our inclination is towards the utilitarian position.

Recently, Camenzind outlined the 3D method, which distinguishes between three dimensions of ethical theory: moral considerability, moral significance, and moral practice (Camenzind 2023). These three dimensions can be seen as successive levels in ethical argumentation, culminating, for example, in the question of how to treat animals, sentient or non-sentient.

The first dimension, *moral considerability*, defines which species are considered morally relevant. For most current welfare definitions/concepts, these are sentient species, a position that reflects sentiocentrism or pathocentrism. Sentiocentrism is the moral position that primarily considers animal sentience and places this aspect at the centre of moral concern. Pathocentrism is a moral standpoint that primarily considers animal suffering as morally significant (Hanlon & Magalhães-Sant’Ana 2014).

*Moral significance* distinguishes between egalitarian and hierarchical variants, adding a second dimension to moral considerability and allowing for a further gradation of moral status (Camenzind 2023).

The central issue of the third dimension, *moral practice*, concerns the content of moral obligations. This issue is addressed by questions such as what are the normative criteria for our moral duties towards beings with moral status, and how to implement respect for the moral status of an animal. Criteria established at this level can be used to formulate guidelines for establishing concrete rules for moral action, i.e. for the appropriate treatment of animals (Schmidt 2011; Camenzind 2023).

In what follows, where relevant, we will attempt to outline the ethical position we have taken with regard to possible solutions to the problem that a large proportion of species in the animal kingdom do not fall within the scope of animal welfare concepts, and that we need guidelines for the appropriate treatment of these species.

## Animal sentience and consciousness

*Sentience* and *consciousness* are ill-defined terms (Veit 2022) and there is no sharp distinction between them. Proctor, for example, stated that *“There is no universally accepted definition of sentience, and there are many different opinions as to where sentience exists in the animal kingdom (…)”* (Proctor *et al.* 2013; p 884). Sentience has been defined in a variety of ways, from definitions that focus on the ability of an animal to experience negative or positive mental states, to definitions that attribute sentience to any living being that responds to environmental influences without the need to experience negative or positive mental states and/or without the need to assume the involvement of conscious processes (for recent discussions that extend to the question whether plants are sentient, see e.g. Calvo *et al.* 2017; Draguhn *et al.* 2021; Segundo-Ortin & Calvo 2022). *“Individuals are sentient if they have the capacity to have feelings, which includes the ability to evaluate the actions of others in relation to oneself and third parties, to remember some of one’s own actions and their consequences, to assess risks and benefits and to have some degree of awareness”* (Broom 2020; p 1). An extreme position is taken by Reber and colleagues (2022), who argue that “*All living organisms are sentient*” (but see, e.g. Draguhn *et al.* 2021; they argue, supported by the results of empirical research, “*that plants do not possess the molecular and structural machinery for pain generation*” (p 246) and the ability to experience pain).

We should keep in mind that *“Evolution tends to be highly conservative when it comes to traits under heavy selective pressure. Sentience and the ability to feel pain are good examples given the inherent fitness benefits”* (Brown 2017; p 3), i.e. it is not necessary to determine the presence or absence of sentience for each group of closely related species, if sufficient scientific evidence has been collected to decide this question for at least one of them (Crump *et al.* 2022). “*The ‘welfare by analogy’ concept suggests that knowledge of welfare in one species can be used to inform us about the welfare of related species (…). The concept assumes that closely related species will have similar needs, for instance, animals that share similar psychological or physiological function, and/or have evolved and adapted to similar ecological pressures”* (Melfi 2009; p 576), a position that is shared by Brown: we even do not need to verify these abilities in each and any species, because *“Closely related taxa tend to share traits through common decent (shared derived characters). Thus, if we know a trait exists in just a few orders within a phylogeny, we can use phylogenetic inference to determine its likely distribution in the phylogeny as a whole”* (Brown 2017; p 3). This angle of vision thus supports the reasonable assumption of sentience in a species that has not yet been deeply investigated, if scientific data confirm sentience in a closely related species.

## A prerequisite for the next steps: Determining whether a species is sentient

Clearly formulated (welfare) concepts should provide the basis for deducing scientific hypotheses/questions that can be addressed using well validated tools, i.e. they should help to identify observables and measurables to answer the scientific questions surrounding the treatment of animals. The “*observables (i.e. elements that can be observed and measured directly)*” (van der Staay *et al.* 2009; p 2), and “*measurables (i.e. elements that can be assigned a qualitative or quantitative attribute)*” (van der Staay *et al.* 2009; p 2) should enable the assessment of an animal’s integrity and/or its welfare (Broom 1991). For any of the putative options to address the problem discussed below, it is crucial to be able to determine whether an animal species is sentient. Assessing their (subjective) emotional state, or level of sentience, will help to take action to improve the treatment of an animal, or to intervene when an animal’s integrity or welfare is threatened or compromised.

To answer these considerations, an appropriate set of well-validated research tools must be available, especially regarding taxa of larger phylogenetic distance from humans (Fiorito *et al.* 2014; Perry & Baciadonna 2017). Crump and colleagues (2022), for example, have recently developed a set of eight criteria for the determination of whether a species is sentient. The more of these criteria are met, the stronger the evidence for sentience. It has been suggested that this set of criteria may need refinement (Brown 2022), and that criteria should be prioritised along the dimension of the strength of evidence for sentience they provide (Irvine 2022). Solms (2022) proposed an alternative set of criteria in response to the list of criteria proposed by Crump *et al*. emphasising the adaptive capacity of an animal species. Both Crump *et al.* (2022) and Solms (2022) agree that animal species may have different levels of consciousness. However, even for (presumably) non-sentient species, we need a clear guide for action. Four possible scenarios are discussed below.

## Putative solutions to the problem that current animal welfare approaches are limited to sentient species

### Formulate alternative concepts, tailored to specific species groups

Separate concepts for different (groups of) species that do not fit the current welfare concepts and therefore lack action guiding knowledge could be formulated. These groups of species share characteristics that can be explicitly addressed by these alternative concepts. Each of these putative bespoke concepts would most likely trigger discussions about the underlying criteria used, and each of the criteria would need careful validation. Vertebrates, including birds, amphibians, fish, and reptiles (in addition to crustaceans and cephalopods) are covered by current welfare concepts and by animal welfare legislation (Simonin & Gavinelli 2019). In these concepts, a crucial component is the ability of an animal to experience negative or positive mental states, at least negative ones such as pain (Vapnek & Chapman 2010). Unfortunately, for a large number of species, it is as yet unknown whether they can experience emotional states.

If a species lacks the capacity to experience negative or positive mental states, and this is most likely to be the case for species with a (very) large phylogenetic distance from humans, then a crucial component of almost all contemporary concepts of animal welfare may not be met. There is a need for alternative concepts to guide action on the treatment of animals for distinct groups of species that do not fall within current sentience-based welfare concepts.

### Apply a ‘welfare’ concept that does not presuppose sentience

The Five Freedoms already recognised a role for emotions in animal welfare, but only for distress and fear, and not for positive emotions (FAWC 1979). In some other early publications on indicators of animal welfare, there was no explicit role for emotional states. For example, in Broom’s early publication (Broom 1986) on animal welfare, there was no mention of sentience and the capacity for negative and positive mental states: good welfare was characterised as an animal being healthy, successfully coping with environmental challenges, reproducing successfully and having a normal growth rate. Nevertheless, the importance of emotional states was explicitly emphasised in subsequent work (Broom 1991). Today, sentience and the ability to experience negative and positive mental states is generally recognised as a crucial component of most welfare concepts.

However, the relevance of sentience in (animal) welfare concepts has recently been debated (e.g. Birch 2022; Bradford 2022). Already half a decade ago, Dawkins (2017), discussed the relevance of consciousness for animal welfare. She defined: *“(…) animal welfare as animals being healthy and having what they want”* (Dawkins 2015; p 31). According to Dawkins, this definition *“(…) avoids paradoxical thinking about animal consciousness and still leaves open the possibility for animal welfare scientists to make major contributions to one of the greatest of all biological puzzles of all – why pain, suffering, and pleasure feel like anything at all”* (Dawkins 2015; p 31). It should be noted, however, that *wanting*, just as *consciousness*, is a product of higher mental processes. Consequently, this concept, similar to those stressing the importance of negative or positive mental states might be unsuited for phylogenetically distant species. It circumvents, however, the problems associated with measuring sentience and consciousness in animals (Dawkins 2022), i.e. we are not urged to reason by analogy, which is easier in species with a short phylogenetic distance from humans (Proctor *et al.* 2013) compared to species with a large phylogenetic distance. Due to the lack of references to sentience, we would not categorise these concepts as welfare concepts, although the authors of these concepts do. That is why we put ‘welfare’ in quotes here.

### Apply the *precautionary* principle

For species at a great phylogenetic distance from humans, there is a huge gap in our knowledge of their level of sentience and whether they are capable of experiencing negative or positive mental states. Applying the precautionary principle has been suggested as an option to guide the treatment of these species (Bradshaw 1999; Martuzzi & Tickner 2004; Croney & Millman 2007; Birch 2017, example: farmed black soldier flies; Barrett *et al.* 2023). According to Manson *“the precautionary principle is supposed to provide guidance with respect to cases in which our scientific knowledge of the harmful effects of a proposed activity is significantly incomplete”* (Manson 2002; p 264), or, as Browning and Veit (2022b; p 6) state *“The precautionary principle advises that in cases of uncertainty we should attempt to err in the interest of caution”.*

Examples in which application of the precautionary principle is considered appropriate are risks associated with the environment (climate change through global warming, acid rain), public health (e.g. potential toxic or teratologic side-effects of new drugs and materials), and exposure to GMOs (genetically modified organisms, such as crops or animals) (Marchant 2001; Manson 2002). Its adoption has also been suggested in relation to the lack of knowledge about the sentience status of insects bred for food production, for example by Delvendahl *et al.* (2022). However, the application of the precautionary principle has been criticised because it can delay or block the introduction of innovations or alternative solutions for which the risks likewise have not yet been fully identified and recorded (Peterson 2007).

Since responsibility must be taken for the consequences associated with the application of the precautionary principle, such as imposing restrictions or taking prophylactic measures, and to avoid its application becoming a *dead end*, proponents of applying the precautionary principle should continually update the available information. Scientists typically offer probabilities and multiple scenarios rather than definitive answers, and extensive research may be required to present findings that provide a clear and unambiguous overview of the issues that threaten the integrity or welfare of an animal and form potential risks of animal abuse (Spruijt *et al.* 2014). The process of filling the knowledge gap that underlies the application of the precautionary principle may involve multiple iterations, i.e. many feedback loops in which new information may trigger new knowledge (Applegate 2002). We agree with Barrett and Fisher (2022) that any welfare concept, or guidance for action, must confront the paucity of data on species with a large phylogenetic distance from humans.

Unfortunately, extensive research may be needed to reach the level of knowledge needed to answer the outstanding questions and make scientifically sound decisions (Spruijt *et al.* 2014). Ultimately, the precautionary principle should be replaced by action guiding knowledge. New insights can lead to changes in the way non-human animals are treated, replacing the precautionary principle with science-based approaches to guide further action (Applegate 2002).

**Figure 2.**
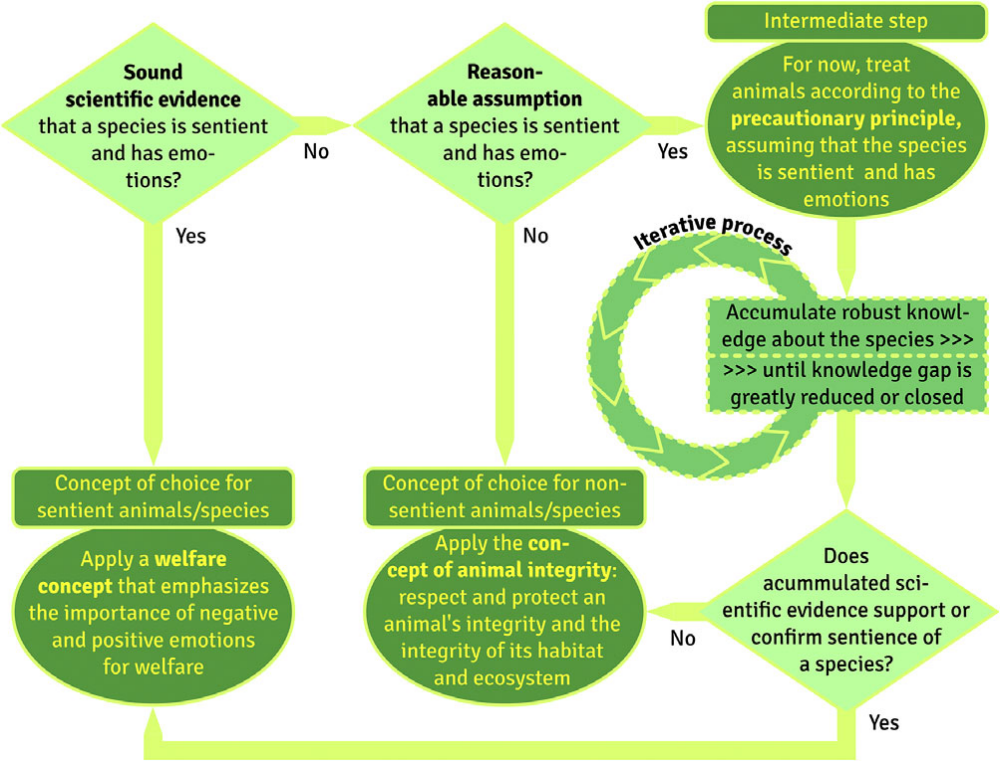
The principle of animal integrity vs welfare concepts.

**Figure 3.**
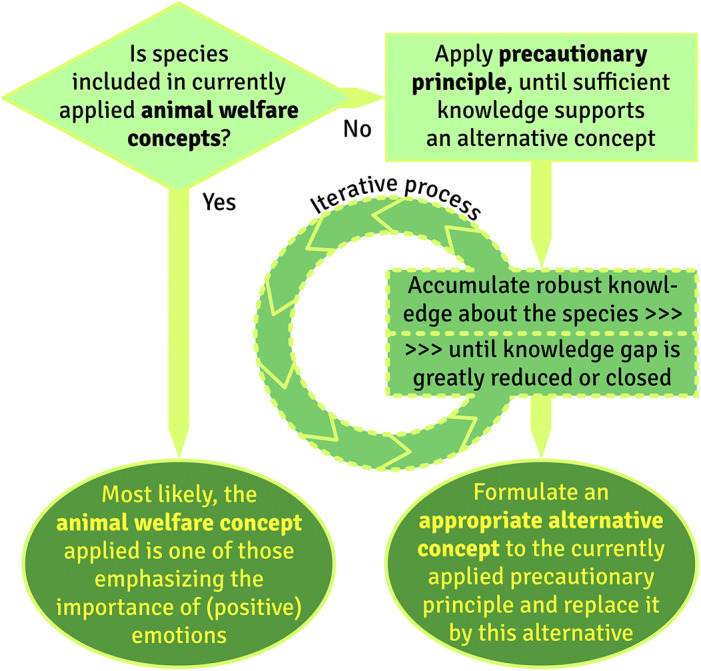
Precautionary principle vs welfare concepts.

Thus, measures taken based on the precautionary principle are tentative, and revisions may become necessary if new knowledge accumulates (see Figure 2). Moreover, one inevitably creates or accepts certain risks when taking decisions about how to treat animal species, as long as the decision to become active or remain inactive are both based on a lack of knowledge (see Figure 3). Finally, the precautionary principle is based on the avoidance of negative consequences, rather than on the creation of positive opportunities, an aspect which is increasingly criticised in previous welfare concepts (e.g. the Five Freedoms).

Thus, instead of or while applying the precautionary principle, one might try to formulate alternatives which allow the generation of testable hypotheses that help to identify observables and measurables to identify problems, apply appropriate solutions and measure whether they have the intended effects.

Scientific research is essential for the development of appropriate strategies for the proper treatment of sentient and non-sentient animals. Empirical research and ethics are interdependent, i.e. empirical evidence informs animal ethicists and their concepts. Welfare can serve as both an empirical and normative criterion (Schmidt 2011) in relation to sentient species. Furthermore, as Webb *et al.* argue, *“biological knowledge on the natural behaviour of different species, in relation to their phylogenetic position and ecology, can help in setting species-specific criteria for animal ethics agendas”* (2019a; p 785). While we do not support the formulation of different (welfare) concepts for different species, we do make a distinction in the accompanying guidance between sentient and non-sentient species, with the former being treated according to current welfare concepts and the latter requiring a different approach, namely that of animal integrity. Röcklinsberg and co-authors see integrity as a concept *“that can bridge what is empirically assessable and what is ethically relevant and experienced at a phenomenological level”* (2014; p 66).

Perhaps the most sensible approach would be to treat animals as if they were sentient or to treat animals based on the reasonable assumption that they are sentient, rather than applying the precautionary principle. Animals that, according to current knowledge, do not have the brain and nervous system necessary to experience emotional states should be excluded from this approach and only if there are reasonable grounds to suspect that they are sentient should species be included. This has been the case with octopods (Low 2012; Mather 2020; Wickens 2022, but see critical comments by Diggles 2019), whose nervous system significantly deviates from that of vertebrates (Hochner 2012).

### Apply the concept of *animal integrity*, eventually expanding to the concept of *habitat/ecosystem integrity*

With regard to the question of who or what should be the subject/object of moral reflection and action, four main approaches can be distinguished. These are *anthropomorphism*, which considers only humans to be morally relevant, *zoocentrism*, in which other animals besides humans are morally relevant, *biocentrism*, which extends moral *relevance* to all living beings, and the all-encompassing *ecocentrism*, which considers all of nature (living and inanimate) to be morally relevant (Hanlon & Magalhães-Sant’Ana 2014). The biocentric and zoocentric views are relevant to our further argumentation. For example, as outlined by Heeger and Brom (2001), Taylor and Rollin, two proponents of these views, argued that animals are *intrinsic goods* and are morally relevant because they are *a good in their own right.*

Whereas Taylor takes a biocentric, egalitarian view, Rollin takes a zoocentric view. Despite his egalitarian view, however, Taylor would deny that different species have to be treated the same. He would claim that they have to be considered equally but treated according to their species-specific needs. Rolling advocates that *“all living beings are equal in having inherent worth does not imply that all are to be treated in the same way. To do so would be inadequate, because living beings differ as to their own good, while realizing its own good is equally important to each living being”* (Heeger & Brom 2001; p 246). Thus, even biocentrists can adopt the view that, although all living things have moral standing, in cases of conflict the interests of some creatures will take precedence over those of others (Humphreys 2014). This implies that different taxonomic classes of animals may require different types and levels of ethical concern (Fraser 1999).

The concept of animal integrity and its elements’ *intrinsic value*, *intrinsic worth* and *good for its own right* are, according to Verhoog (2000), central to the biocentric theory (Humphreys 2014). They lead to normative obligations regarding our actions towards and interactions with animals. The concept of integrity is also sometimes used in relation to species and ecosystems. Leopold, for example, suggested that ethical obligations can be derived from the fact that humans are part of and have a relationship with their ecosystem (see Beatley 2014). In this paper, we adopt the perspectives described in this section.

In contrast to current definitions of the concept of animal welfare, in which the animal’s *subjective* experience (whether positive or negative) is a crucial component, there is no such role for subjective experience in the concept of animal integrity. The integrity of the animal can be in jeopardy without the animal being *aware* of it (Vorstenbosch 1993; p 111). The most commonly used and cited definition of animal integrity, i.e. the classic definition, comes from Rutgers and Heeger (1999). They defined animal integrity as *“the wholeness and completeness of the animal and the species-specific balance of the creature, as well as the ability of the animal to sustain itself independently in a species-appropriate environment (…)”.* (Rutgers & Heeger 1999; p 45). The three components of their definition are interrelated and complementary and must be met to ensure the integrity of an animal (Rutgers & Heeger 1999). In a number of recent publications, the integrity of an animal’s genome is discussed (e.g. Bovenkerk & Nijland 2017; Bovenkerk 2020).

Thus, in addition to internal factors (such as an animal’s state of health and genetic constitution), the external environment and ecological factors contribute significantly to animal integrity. The environment and living conditions of all animals must meet their biological requirements and behavioural needs. This means that ecological factors and behavioural characteristics should be taken into account. These factors can be scientifically studied and quantified, and findings could fill knowledge gaps (see above) and provide guidance for the treatment of these animals, requiring respect for their behavioural needs and protection of the integrity of their habitat (see Figure 2).

More explicitly than the *precautionary principle*, the concept of *animal integrity* considers the conditions that are critical to an animal’s ability to maintain its own health, survival and reproduction. Avoiding application of the *precautionary principles* and embracing the concept of *animal integrity*, two putative options for treating animals emerge, with one additional intermediate alternative that should be followed as long as the evidence base is insufficient to choose one of the two final options (Figure 2): *Apply a welfare concept* or *Apply the concept of animal integrity.* It is the aim of this decision process to base all actions on a sound scientific basis, depending on whether a species is sentient and able to experience negative or positive mental states, or not. Note that even sentient species are thought to go through a period of development during which they are not yet capable of conscious perception. For young mammals and birds, this is the period before birth or hatching. Due to this lack of conscious perception, their welfare is not at stake during this period (Green & Mellor 2011).

Combining the wording of Rutgers and Heeger’s (1999) definition of animal integrity with our Dynamic Animal Welfare Concept (DAWCon; Arndt *et al.* 2022), we propose this definition of animal integrity:

*An animal’s integrity is most likely to be intact when its wholeness, its species-specific balance and its ability to sustain itself independently in an environment appropriate to its species are ensured, i.e. when its environment provides the resources necessary for survival and reproduction, and when the animal has the ability and opportunity to cope appropriately with the challenges of the (prevailing) environmental circumstances.*

This definition can be applied to all animal species (see the definition of *animal* in the first paragraph of the *Introduction*), regardless of their phylogenetic distance from humans, and does not refer to the animal’s internal state (e.g. emotions). Other authors adapt and apply integrity not only to the individual animal, but also to the environment and ecosystem – habitat or ecosystem integrity – in which he or she lives. (Vorstenbosch 1993; Bovenkerk *et al.* 2002). This definition also refers to the role of humans and their responsibilities towards all living creatures and their habitats, i.e. this concept of animal integrity includes the *duty of care* (Mellor & Stafford 2001; Ohl & Putman 2014; Weary & Robbins 2019; Learmonth 2020) and the *duty to protect the habitat and ecosystem* of these species (see also Díaz *et al.* 2019). The importance of the latter is that an intact environment is more likely to allow an animal to meet its biological needs and, in the case of sentient animals, to achieve a state of positive welfare.

The dynamic nature of animal welfare, operating on a continuum between good and bad welfare states, is captured by theoretical concepts of animal integrity, as well. *“Integrity is not only a state in which living beings can find themselves, the concept also includes a specific ability to integrate. The organism itself is actively involved in the process of creating – maintaining and, if necessary, restoring – its own integrity. It controls and regulates the finely tuned interaction of the organism as a whole with its individual parts and its environment.”* (Schmidt 2008; p 318, translated from German). Evolution has equipped animals with the means to cope with challenges of the environment in which they evolved, i.e. both the animals’ integrity as well as that of their environment and ecosystem should be respected and protected.

## Discussion

The acknowledgment of the potential to suffer in mammals (e.g. DeGrazia & Rowan 1991), has led to the increasing use of species with a larger phylogenetic distance to mammalian species, such as fish (e.g. Sneddon 2004; Lidster *et al.* 2017) and invertebrates (Andrews 2011; Huber et al. 2011; Wilson-Sanders 2011; Adamski *et al.* 2019) in scientific research. For species (or their developmental stages; Mellor 2019) that (may) lack this ability, current welfare concepts, based on sentience and experiencing emotions (at least of feeling pain) are inadequate. For species kept for our own purposes (e.g. farmed insects), we have moral obligations and responsibilities based on the biocentric viewpoint. These obligations can thus be translated into the formulation of guidelines for action based on ethical and moral principles that recognise the integrity of the animal (Fraser *et al.* 1997; Christiansen & Sandøe 2000).

We have presented four different options for animal species that are not sentient or for whom it is not yet known whether they are sentient. Of these, we favour the option of applying the concepts of animal integrity (Rutgers & Heeger 1999) and the integrity of the animal’s habitat/ecosystem (Bovenkerk *et al.* 2002), which go beyond current welfare concepts for sentient species. While the integrity of the animal is directly related to its unimpaired biological function, the integrity of the ecosystem may be a prerequisite for the animal to maintain this function. They can be used in addition to, rather than as a substitute for, current welfare approaches for sentient species, to derive guides for action.

### Four putative options to address the problem that current animal welfare approaches are limited to sentient species

#### Option 1 – Formulate alternative concepts, tailored to specific species groups

This is an obvious option as it could take into account the specificities of different species. However, each individual definition would lead to a multitude of discussions about the criteria used and their validity, as is already the case with discussions about current welfare concepts. In addition, it is not clear how many different definitions would be needed in order to establish relevant guidelines for action for all species of animals. This approach could therefore lead to a never-ending stream of new concepts to guide the treatment of these species. Instead of adopting concepts that are specific to particular (groups of) species, one could adopt a concept that is generally applicable to all non-sentient animal species. Three such options are outlined in what follows.

#### Option 2 – Apply a ‘welfare’ concept that does not presuppose *sentience*

The *sentience-free* or *consciousness-free* concept, but otherwise similar to those welfare concepts that emphasise the importance of negative or positive mental states proposed by Dawkins (2015), may be inappropriate for phylogenetically distant species. It focuses on what an animal *wants*, which requires the presence of higher mental functions, although it avoids the problems associated with measuring sentience and consciousness in animals. Thus, we are not asked to reason by analogy, which is easier for species with a short phylogenetic distance from humans (Proctor *et al.* 2013) than for species with a large phylogenetic distance. The *consciousness-free welfare concept* avoids discussions about the question of whether we can measure sentience in animals scientifically and, if that question is answered in the affirmative, how we should measure it (Dawkins 2006; Birch et al. 2022; Browning & Veit 2022a; Solms 2022). However, a *consciousness-free welfare concept* does not take into account the animal’s perception of its own mental state, which is explicitly addressed in other current animal welfare concepts (see, e.g. “*an animal has good welfare when it reaches a state that it perceives as positive, i.e. that evokes positive emotions*”; Arndt *et al.* 2022; p 3, and similar formulations in other welfare concepts). This concept therefore misses a crucial aspect of an animal’s welfare.

#### Option 3 – Apply the *Precautionary principle*

If the precautionary principle is not supplemented by continued efforts to close the knowledge gap scientifically, it puts the brakes on furthering our understanding and maintains the application of restrictions and actions which, though well intended, might be ineffective or less effective than those based on relevant sound scientific knowledge. Consequently, the aim should be to replace the precautionary principle by knowledge-based measures as fast as possible, driven by relevant scientific research.

Application of the *precautionary principle* is bound to the restriction that the measures taken should be *cost-effective*, a condition that is also a requirement in the legal version of this concept. To decide whether a measure is *cost-effective* would require a cost-benefit, harm-benefit, or a cost-harm analysis, i.e. an analysis that balances the interests of non-human animals and humans. Such analyses are usually performed before introducing new housing conditions and management procedures for farm animals that intend to improve animal welfare (Fernandes *et al.* 2021), and during the approval process of experimental animal research (Brønstad *et al.* 2016). However, these analyses are hampered by a lack of knowledge about species with a large phylogenetic distance from humans, who are the primary target group for the application of the precautionary principle.

Critics of this view point out that the concepts of *precautionary principle* and *precautionary measures* are ambiguous and ill-defined (Marchant 2001; Manson 2002; Turner & Hartzell 2004). There are more reasons to doubt that the precautionary principle is appropriate as an alternative to current welfare concepts for animals with large phylogenetic distance from humans. We might, for example, ask what kind of *risk* and what *harmful effects* are tackled by applying this principle. With respect to animal welfare and their quality of life, the risks are: doing harm (causing pain, distress) to animals who are sentient, whereas we assume they are not, neglecting threats of serious, negative animal welfare outcomes as consequence of our actions (Birch 2017), or disturbing the balance of the ecosystem if we endanger a species (destruction of its habitat) (Marchant 2001; Manson 2002). The effects of the consequences associated with the latter risk may extend to other living beings, including humans.

The precautionary principle is being applied because of fears to compromise welfare or the quality of life of animal species about whom insufficient knowledge has accumulated or because of uncertainty about consequences of becoming active or remaining inactive in view of a putative risk. The weakest aspect may be that we take precautionary measures based on intuition or on applying the principle of analogy (e.g. Sherwin 2001) because a proper risk analysis is impossible due to a lack of hard evidence and knowledge and/or of appropriate research tools. Even more, uncertainty is a prerequisite of the precautionary principle and why this principle is applied in the first place. A species should be treated according to the precautionary principle only if one condition is met, namely that there is a reasonable presumption that the species is sentient (for example, because phylogenetically close species have already been scientifically classified as sentient) (see also Figure 2).

#### Option 4 – Apply the concept of *animal integrity*, eventually expanding to the concept of *habitat/ecosystem integrity*

We prefer to apply the concept of animal integrity to all animal species. Additional species for whom there is accumulating scientific evidence that they can be considered ‘sentient’, i.e. capable of experiencing positive and/or negative mental states, will be added to the list of species covered by the concept of animal welfare. Thus, in the long term, this approach will lead to a broadening of the range of species to whom the concept of animal welfare applies. The concepts of animal welfare and animal integrity apply to all sentient species, while non-sentient species fall under the concept of animal integrity. However, the adoption of the principle of *animal integrity* raises new questions that must be addressed, such as:

- What threatens the animal’s quality of life or even the existence of the animal/species, and what is necessary to provide the animal with living conditions in which he or she can thrive? This aspect is closely linked to habitat/ecosystem integrity.
- What does an animal want and what does he or she do if given a free choice (Franks 2019)?

These questions can be answered scientifically by studying the ecology and behavioural biology of animal species and individuals within species. The necessary techniques and methods are available and/or can be developed. In addition to this basic scientific approach, ethical questions remain, such as:

- Whose interests prevail when they conflict with those of other species (including humans), e.g. in the case of animals who are considered pests, animals who may be dangerous to humans or other animals, or who lay claim to the same resources (a recent example is the discussion/controversy about the return of the wolf and other large carnivores to Europe; Trouwborst 2010; Breyne *et al.* 2021). This relates to the question of moral status, i.e. whether moral responsibility is the same for all groups of animals, or whether there is a gradation of moral status that allows for differential treatment (see also dimension 2 in the 3D method by Camenzind 2023).
- Under what conditions and to what extent are humans responsible for the welfare/integrity of animals? This is a question related to the principle of *duty of care* (Ohl & Putman 2014).

### Future directions

As sentience is a core element of most, if not all, recent welfare concepts, we suggest further study of neural morphology and function (e.g. Roth II *et al.* 2019), ideally in a comparative framework, encompassing a wide taxonomic range of species. A recent example is a systematic review by Miller *et al.* (2022) in which cognition and welfare is compared across a broad range of ten farmed taxa, using a broad range of criteria.

Promising developments have been made to assess (welfare) states of animals: cognitive bias and attention bias tests, for example, have the potential to assess the effects of internal and external factors inducing both positive and negative (behavioural and physiological) consequences in a broad range of species (from insects to humans) (e.g. Mendl *et al.* 2009; Murphy *et al.* 2014; Crump *et al.* 2018). Nevertheless, further cross-species validation and careful interpretation are required (Roelofs *et al.* 2016). The differences in response to positive or negative stimuli between species (and individuals), and the physiological and behavioural consequences of this, imply that these should be taken into account, irrespective of the species’ capacity to experience negative or positive mental states. The biological foundations of emotions, affect and consciousness need to be further investigated. How to differentiate between reflexes and consciously controlled behaviours is one example of a research question.

Deprivation of the possibility to perform natural behaviour has been recognised by several welfare concepts as a factor leading to poor welfare (Dawkins 1988; Bracke & Hopster 2006). However, the often-stated view that natural behaviour is an important aspect of welfare has also been criticised (e.g. Browning 2019; Arndt *et al.* 2022; Dawkins 2023). For many species, both wild and captive, our knowledge about their natural behaviour is still incomplete, fragmentary or, in the worst cases, non-existent. Knowledge about the behavioural repertoire of species needs to be gathered, including but not limited to the compilation of comprehensive ethograms, including sex- and age-specific behaviours. Information on physiological requirements, ecology (of wild ancestral species) and health indicators should also be studied and documented. Researchers could share their experiences of husbandry and positive or negative results in a common database. In particular, empirical evidence for furthering our understanding of *good* welfare or integrity in different, in particular in phylogenetically distant taxonomic classes, is urgently needed. In some species, (positive) emotions surely are mandatory to reside in a state of positive welfare. However, the very basic problem, that conclusions on animal emotion or affect can only be drawn based on humans as reference, remains, and this problem will be unsolvable as long as emotion and affect are not better understood in humans (Gutfreund 2017).

The lower the level of analogy with humans the less solid is the (scientific) basis for the presumed presence of affective states and the potential to suffer, in a species. Should evidence accumulate that supports the notion that some species with larger phylogenetic distance from humans lack the ability to experience negative and/or positive affect, we may need a guide for action concerning the treatment of these species. The fact that we are currently unable to answer the question of whether we need specific concepts to guide action for different taxonomic classes does not relieve us of the obligation to anticipate possible impairments of the species’ adaptive capacity and integrity, and to take measures to prevent putative negative effects. Or, as Gutfreund recommended, *“Animals should be treated with respect and compassion because this is the most sensible and humane thing to do, irrespective of findings emerging from laboratories studying animal brains and behaviors”* (Gutfreund 2017; p 199).

### Animal welfare implications

The approach we propose offers a conceptual basis for the development of appropriate guidelines and policies to ensure the integrity and, in the case of sentient species, the welfare of animals. We argue that the concept of *animal integrity* should be applied to all animal species, whether or not they fall under current animal welfare concepts, i.e. whether or not they are sentient and capable of experiencing (positive) emotions. The concept of animal integrity should be applied in addition to, rather than as a substitute for, current welfare approaches for sentient species to derive guides for action.

## Conclusion

The integrity of the animal and the provision of opportunities to meet its behavioural and physiological needs should guide the treatment of any species kept for human purposes. The discussion of animal welfare and animal integrity has a strong ethical component that goes beyond a purely biological perspective. This moral aspect of concepts of animal welfare may explain why this discussion is perhaps currently more alive among philosophers and ethicists than among animal scientists and neuroscientists. The 3D methods recently outlined by Camenzind (2023) can be used to analyse and determine the ethical/moral position taken with regard to: (1) moral considerability (i.e. who counts morally?); (2) moral significance (i.e. how much does the animal count?); and (3) moral practice (i.e. how should an animal be treated given its moral status?). Further discussion will be needed before all these questions can be answered satisfactorily. Looking at the questions raised from the perspective of our expertise in behavioural biology, biopsychology and animal science, we have made a first attempt to answer at least some of them. Further discussion of these issues should involve experts from all relevant disciplines.

**Figure 4.**
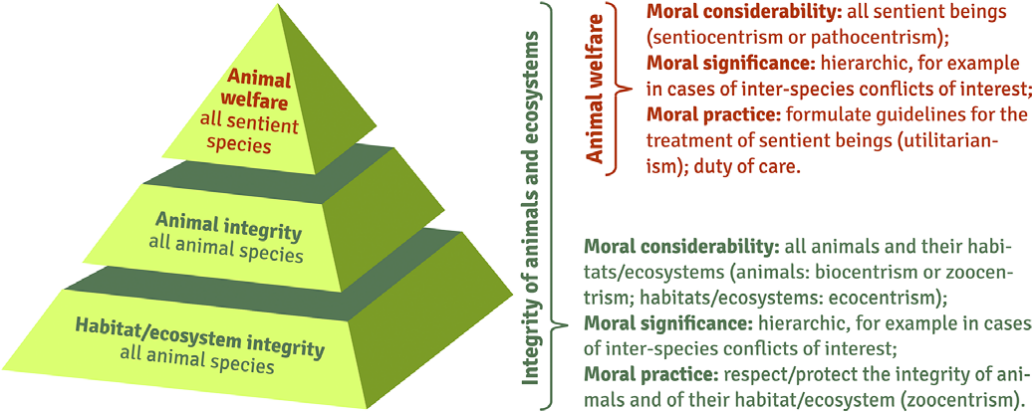
Hierarchy of concepts (animal welfare, animal integrity, ecosystem integrity).

Clear guides are needed for all animal species, not just those covered by current animal welfare concepts. We propose to apply the concept of *animal integrity*, possibly complemented and extended by the concept of *habitat/ecosystem integrity*, to all animal species. For species that are sentient and capable of experiencing (positive) emotions, a current concept of animal welfare should serve as the primary basis for guidance on how to deal with them (see Figure 4), i.e. it should be applied in addition to the principle of animal and ecosystem integrity.
